# Development of an innovative physical examination course involving handheld ultrasound devices

**DOI:** 10.15694/mep.2018.0000164.2

**Published:** 2019-01-07

**Authors:** Achim Jerg, Michael Denkinger, Lucia Jerg-Bretzke

**Affiliations:** 1 University Hospital Ulm; 2 University Hospital Ulm; 3 AGAPLESION Bethesda Clinic Ulm; 4 AGAPLESION Bethesda Clinic Ulm

**Keywords:** Clinical Skills, Handheld Ultrasound, Physical Examination, Practical Training

## Abstract

This article was migrated. The article was marked as recommended.

Several studies in recent years have shown that the physical examination skills of medical students are inadequate. In response to this deficit, a new teaching intervention has been developed consisting of five physical examination courses and a set of corresponding bedside teaching modules. The bedside modules are primarily intended to provide the opportunity for practical application of the examination techniques learned. One particularity of the bedside teaching was the use of handheld ultrasound (HHU) units in order to be able to visualize and verify/falsify diagnostic findings immediately. Since this demonstration of findings was standardized according to the specifications of the Rapid Ultrasound in Shock and Hypotension (RUSH) protocol, it constituted the basis for the communication of basic emergency ultrasound skills. A pilot study, which included an initial evaluation, has demonstrated this concept is feasible and is met with great interest on the part of the students.

## Introduction

Together with the taking of a patient’s medical history, the physical examination constitutes the basis of any medical treatment. This statement is supported by the fact that over 75 percent of all diagnoses can be made based solely on the medical history and physical examination ( Peterson *et al.*, 1992; Reilly, 2003). Despite this, the training of junior physicians in practical physical examination skills has been neglected for years. The reasons for this are manifold. One very important aspect is the lack of suitable lecturers and teaching time for bedside examinations over the course of a student’s medical studies ( Ramani *et al.*, 2010; Smith, Burton and Mackay, 2009). Unfortunately, there are few opportunities after graduation to make up for the failure to acquire such skills. For example, junior physicians are rarely supervised when performing physical examinations and thus receive little instruction that could lead them to optimize their skills ( Burdick and Schoffstall, 1995; Sachdeva *et al.*, 1995; Stone, Angevine and Sivertson, 1989). This is made even more difficult by their performance of intensified chart rounds and reduced time in the actual patient’s room ( Collins, Cassie and Daggett, 1978; Shankel and Mazzaferri, 1986). A U.S. study found, for example, that approximately 36 percent of physicians at an academic teaching hospital no longer carried out any physical examinations during their rounds ( Smith, Gertler and Freeman, 2003). The physicians’ faith in technology, time pressure, and shorter patient stays further aggravate the problem ( Dunnington *et al.*, 1992; Hardman, Patel and Delbridge, 1997; Leese and Bosanquet, 1996; Scott and Wordsworth, 1999). Ultimately, this situation results in a vicious cycle because the frequency of physical examinations is decreasing, therefore the importance of physical examinations is underestimated, and finally coverage of physical examination techniques during medical degree studies is being neglected to an increasing extent. It is therefore hardly surprising that countless scientific papers mercilessly address physicians’ deficits regarding physical examinations ( Mangione, 2001; Mangione and Nieman, 1999; St Clair *et al.*, 1992; Vukanovic-Criley *et al.*, 2006).

## Objective

An examination course employing a new didactic approach was developed in order to counteract the decreasing significance of the physical examination. The goal of this course is to improve clinical examination skills through intensive, practical teaching methods. In addition, the idea is to allow for the visualization of findings by way of immediate verification/falsification. To facilitate this, handheld ultrasound (HHU) devices were integrated into the course. Although the physical examination is the core of the teaching intervention, the inclusion of the HHU will serve to simultaneously convey emergency ultrasound skills based on the RUSH protocol (Rapid Ultrasound in Shock and Hypotension; see 2.2) ( Seif *et al.*, 2012).

## Teaching intervention

The teaching intervention consists of five physical examination courses and subsequent bedside teaching modules using HHU devices (e.g. GE Healthcare VScan®, ca 8.000 USD). While the examination course is intended to convey initial examination skills, the bedside teaching module focuses on the practical application of the knowledge acquired. HHU devices are used to visualize and verify the medical findings arrived at during the bedside teaching module. In addition, students will learn the basics of emergency ultrasound as detailed in the RUSH protocol ( Seif et al., 2012). In order to ensure that the course ran on schedule, the teachers were given a one-hour technical briefing on the HHU devices in advance. A clinic employee was responsible for the maintenance of the devices and for resolving any technical queries.

### Physical examination course

The teaching intervention comprises five examination courses with a focus on internal medicine. The substantive basis of the courses is a catalog of learning objectives (see Table 1) based on a study by Haring et al. ( Haring, van der Meer, Jos W M and Postma, 2013), which defined the examination skills to be mastered by medical students. As part of the five courses, the examination techniques thus identified will be taught primarily through partner exercises under the guidance of specialist physicians. The first examination course focuses on refreshing the skills acquired thus far. In addition, students must master the correct technical execution of inspection, palpation, percussion, and auscultation, as well as the formulation of medical findings. The first examination course is therefore the basis of the subsequent courses: “Heart and vessels,” “Lungs and thorax,” “Abdomen,” and “Emergency examination.” Regardless of the content, the sequence of the physical examination courses is always the same and can be divided into demonstration, deconstruction, comprehension, and implementation - following a modified version of Peyton’s technique ( Krautter et al., 2011; Krautter et al., 2015). Initially, the examination techniques are demonstrated in their usual sequence and at the regular pace by the physician teaching the course (demonstration). Subsequently, the examination technique to be taught is divided into individual, easy-to-understand actions (deconstruction). To check whether the content taught has actually been understood, the teacher then asks the medical students to describe the individual steps while he/she carries them out - in a sense, the instructor is now being instructed by the medical students. This promotes an accurate mental representation of the procedure and allows for faster learning and improved recall (comprehension). The newly-acquired skills are then applied to the patient in practice as part of the bedside teaching module, using HHU devices (see 3.2).

### Bedside teaching

The bedside teaching module is divided into two didactic elements: first, the transfer of the skills taught during the examination courses to the patient’s bedside and second, the teaching of the basics of HHU devices as detailed in the RUSH protocol (see Table 1) ( Seif et al., 2012). The bedside teaching module is also based on demonstration, deconstruction, comprehension, and implementation following a modified version of Peyton’s technique ( Krautter et al., 2011; Krautter et al., 2015). Strictly speaking, this only applies to ultrasound training according to RUSH. The focus with regard to the physical examination is solely on the implementation.

Following the physical examination course, the students are divided into small groups of up to four people and taken to selected patients. The patients are selected by the instructors in advance, ensuring that a pathology in line with the examination course should be detectable. For example, a patient with ascites or the like will be selected for the “Abdomen” physical examination course. Of course, patient consent is obtained. After the students and the patient have been introduced to each other, the newly-learned examination techniques are applied at the patient’s bedside under specialist supervision and medical findings formulated. If any problems occur during the practical implementation, the students are corrected immediately by the instructor. In the case of the example chosen for the “Abdomen” bedside teaching module, the students examine the patient as they have learned during the physical examination course and identify a dampening of the sound while performing a percussion on both sides of the body, for example. They verbalize this finding and make the suspected diagnosis of ascites. At this point, the examination transitions to the HHU: first, the medical finding is verified/falsified by the instructor using a HHU device. This is also the first step of the demonstration following Peyton’s technique. What is important in this context is that the findings are not simply presented, but that - similarly to the physical examination - all ultrasound steps are demonstrated in their usual sequence and at the regular pace. In the case of the abdomen, this includes examination for free fluid in the hepatorenal recess (Morison’s pouch), splenorenal recess (Koller’s pouch), and the dorsal side of the bladder, as well as the presentation of the aorta caudal to the heart, infrarenal, and before the bifurcation (see Table 1). Subsequently, the ultrasound skills to be taught are divided into easy-to-understand steps by the instructor and demonstrated again (deconstruction). Then, the students are asked to instruct the instructor with regard to the practical implementation of the steps so as to test their understanding of the process. Finally, the students are actively challenged to implement the learned steps of the ultrasound examination independently. As in the case of the physical examination, implementation and possible correction take place under the supervision of an instructor.

**Table 1.** Overview of the learning objectives related to physical examination and ultrasound taught in the examination courses. HHU: hand-held ultrasound, RUSH: Rapid Ultrasound in Shock and Hypotension, VOR: vestibulo-ocular reflex

Physical Examination Course	Examination techniques	HHU learning objectives (RUSH)
Introduction Examination techniques and findings Head and neck	Examination procedure, preparation, and scheme Correct technical implementation of inspection, palpation, percussion, and auscultation, as well as formulation of medical findings Skin and skin turgor Inspection of eyes, oral cavity, and cervical veins Palpation of regional lymph nodes, thyroid, and frontal and paranasal sinuses	Physical principles of ultrasound Examination procedure according to the RUSH protocol ( Seif et al., 2012)
Heart and vessels	Heart: Inspection and palpation of the apex beat Percussion of boundaries of the heart Auscultation of the heart Vessels: Palpation of the carotids, abdominal aorta, femoral artery, popliteal artery, dorsalis pedis artery, and posterior tibial artery Auscultation of the carotids and the femoral artery	Visual assessment of left ventricular pump function Hypertrophy of right ventricle, signs of right ventricular strain Pericardial effusion Valvular dysfunction or restricted valvular function Wall motion abnormalities Estimation of the volume status by measuring the inferior vena cava (IVC)
Abdomen	Inspection of abdomen and groin Superficial and deep palpation of the liver, spleen, kidneys, and inguinal lymph nodes Percussion of liver, spleen, abdomen, bladder Auscultation of the abdomen	Free fluid in the hepatorenal recess (Morison’s pouch), splenorenal recess (Koller’s pouch), and the dorsal side of the bladder (Douglas) Presentation of the aorta caudal to the heart, infrarenal, and before the bifurcation
Lungs and thorax	Inspection of thorax and breasts Palpation of breasts and axillary lymph nodes Percussion of lung fields and dampened sound during percussion of the diaphragm Auscultation of the lungs	Pleural effusion, hematothorax Pneumothorax
Emergency examination	A - Check for airway obstruction B - Respiratory rate, lung auscultation C - Blood pressure, pulse rate, cardiac auscultation, capillary reperfusion time D - Babinski and pupil reflexes, VOR E - Body temperature	Independent performance of the RUSH examination (target: maximum of five minutes)

### Practical implementation

The concept presented here can be implemented in a number of ways, for example in the context of existing clinical examination curricula. The course could also be established as an elective, as part of summer/winter school, as a seminar for entrants into the labor market or integrated into clinical internships or traineeships.

As a model project, the teaching intervention has hitherto been implemented as an elective as part of a four-week clinical traineeship with ten participants. Each week, a correlated block consisting of examination course and bedside teaching module was completed, with the first teaching unit delivered just prior to the start of the clinical traineeship due to its introductory nature. The last examination course, “Emergency examination,” served as a revision course and a forum for discussing any remaining questions. The exam for the elective was administered during the subsequent bedside teaching module in the form of a Mini Clinical Examination (Mini-CEX).

## Evaluation

During the piloting phase, which involved ten participants, the teaching intervention proved to be a feasible teaching format from an organizational standpoint. The keen interest of the medical students has encouraged us to continue the project and demonstrates the need for the teaching format used. In addition, the students who participated were asked to complete a short questionnaire following the teaching intervention. This questionnaire confronted the participants with statements regarding the teaching intervention that were to be answered with Yes or No. Although it is not possible to draw any statistically relevant conclusions due to the small number of participants, the initial evaluation indicated that the format used could be beneficial. Almost 90 percent of the students participating stated after completing the training intervention that they felt more secure when conducting physical examinations thanks to the teaching format. An equally large percentage of students described the use of HHU devices as helpful in improving their examination skills. The answers to the question of whether they would recommend participation in the teaching intervention were particularly motivating, since all participants answered in the affirmative. In addition to the questionnaire, individual interviews were also sought with the participants. Many criticized the small screen size of the HHU devices, which makes the detection of pathologies difficult for the inexperienced. One possible solution could be to record relevant sequences. These could then be better explained using a projector during a debriefing. After a short introductory session, the HHU devices were perceived as easy to operate. No technical problems occurred over the course of the pilot project. Some students suggested that a script should be created to accompany the tutorial for the purposes of individual preparation and follow-up on the examination techniques taught.

## Discussion

The number of participants surveyed as part of the pilot project was too small for a quantitative analysis with regard to the possible benefits of the teaching intervention. Therefore, the new teaching format must be evaluated using a larger sample. Nevertheless, the statements of the medical students who participated show that the objectives of the teaching intervention may have been achieved. The procedure described ensures immediate feedback regarding the technical implementation of the examination techniques and also offers a comprehensible review of the medical findings. Both factors together result in improved examination skills ( Dunnington et al., 1992). Another advantage of the bedside teaching module is the realistic environment, which is crucial for lasting learning achievements ( Brown, Collins and Duguid, 1989; Monti et al., 1998). In addition, this form of teaching and learning is assessed most positively by students ( Celenza et al., 2001). Learning the basics of emergency ultrasound examinations also appears realistic using this method ( Blackstock, Munson and Szyld, 2015; Fernández-Frackelton et al., 2007; Hoppmann et al., 2011). One limitation that should be mentioned at this point is the fact that the recruitment of patients with suitable pathologies is challenging and will not always be successful. It is possible that the observations mentioned above are not due to the use of HHU, but rather a result of the dedication and commitment of the lecturers. Since this may not always be guaranteed, the teaching intervention could also be carried out in the peer teaching format. This would allow the project to be scaled and expanded further. There is evidence from different educational settings that peer teaching benefits both the peer teachers and the learners. This suggests that peer teaching could be a valuable methodology for medical schools to improve teaching in physical examination skills and HHU ( Benè and Bergus, 2014). Ultimately, only a more extensive study can show the potential benefits of this teaching intervention with regard to physical examination skills and RUSH. Due to the extremely positive reception of the method among students, such a study is currently being planned.

## Conclusion

The teaching intervention proved to be a feasible format which found great acceptance among students. Although the evaluation performed thus far can only reveal rough trends, there is reason to believe that the project in question can improve the physical examination skills of medical students. In addition, the integration of HHU devices into the teaching intervention could not only facilitate the visualization and verification of medical findings, but also the acquisition of basic bedside emergency ultrasound skills in accordance with the RUSH protocol.

## Take Home Messages

• Medical students have severe deficits in performing physical examination (PE).
• To improve students`s PE-skills an innovative course involving handheld ultrasound (HHU) was implemented as a pilot project.
• The integration of HHU devices into the teaching intervention could not only facilitate the visualization and verification of medical findings, but also the acquisition of basic bedside emergency ultrasound skills (RUSH protocol).
• Feasible format with great acceptance among students.

## Notes On Contributors

Achim Jerg studied Biology and Medicine at Ulm University, Germany. In his doctoral thesis - which was rated as summa cum laude - he developed a new teaching method for improving medical students` physical examination skills. Currently he works as an Internal Medicine resident.

Michael Denkinger studied Medicine at University of Freiburg, Germany. After finishing his studies he worked as a scientist in Cleveland, USA. In 2011 he became senior physician in Internal and Geriatric Medicine and in 2014 head physician. Since 2012 he is professor of Internal and Geriatric Medicine at Ulm University.

Lucia Jerg-Bretzke studied Economics at University of Augsburg. After working in private economy she switched to Department of Psychosomatic Medicine and Psychotherapy at the University Hospital Ulm. There she works as a scientist in fields of working conditions and their psychosocial impact.
