# Supplementary material for: Development of an innovative physical examination course involving handheld ultrasound devices
Source: MedEdPublish (2016). 2019 Jan 7;7:164. Originally published 2018 Aug 7. [Version 2] doi: 10.15694/mep.2018.0000164.2 (PMC10701831; doi:10.15694/mep.2018.0000164.2)
Supplement: Peer review history

# Reviewer report: Reviewer response for version 2

**DOI:** 10.21956/mep.20225.r31324

**Published:** 2019-01-13

**Authors:** Susmita Reddy Karri

**Affiliations:** 1 Topiwala national medical college and BYL nair charitable hospital, mumbai. india

This review has been migrated. The reviewer awarded 4 stars out of 5 This paper throws light on one the basic, but the most important skill that differentiates a good from a bad physician: examination. A very innovative idea indeed. Maybe we could finish off the loop by reassessing the above students.

Reviewer Expertise:

NA

No decision status is available

# Reviewer report: Reviewer response for version 2

**DOI:** 10.21956/mep.20225.r31326

**Published:** 2019-01-11

**Authors:** Balakrishnan Nair

**Affiliations:** 1 Centre for Medical Professional Development and University of Newcastle

This review has been migrated. The reviewer awarded 4 stars out of 5 History and physical examination will provide the diagnoses in majority of cases. This will help the physician to order targeted and cost effective investigations. The investigations then should help to fine tune the diagnostic skills of the physician . ( A good example is a heart murmur and cardia echo). In spite of this bedside examination is a dying art. It is good to see this research on the teaching of bedside examination with the US examination to fine tune the clinical examination skills . This will give immediate feedback to the learner .It is good to see the clinical examinations were deconstructed into simple stepsHowever there were only 10 students in the study. It would be good to see how the students performed in the physical examination ( eg-cardiac apex and accuracy of the LV size on the US) . Another potential of the study will be to reassess the students to see whether the skills were retained. I congratulate the authors for this excellent work and would love to see the follow up papers .

Reviewer Expertise:

NA

No decision status is available

# Reviewer report: Reviewer response for version 2

**DOI:** 10.21956/mep.20225.r31325

**Published:** 2019-01-07

**Authors:** Felix Silwimba

**Affiliations:** 1 University of Lusaka

This review has been migrated. The reviewer awarded 5 stars out of 5 this clinical medical education study report is clear and illustrative. it has addressed a threatened skill of the art of a physician. Diagnostic medical technologies should still be treated as disruptive. Physical examination remains an important skill not to be replaced by technology or AI

Reviewer Expertise:

NA

No decision status is available

# Reviewer report: Reviewer response for version 1

**DOI:** 10.21956/mep.18983.r27203

**Published:** 2018-08-12

**Authors:** Trevor Gibbs

**Affiliations:** 1 AMEE

This review has been migrated. The reviewer awarded 2 stars out of 5 An interesting paper although I am not sure that the solution of using hand-held ultrasound equipment solves the problem related to clinical examinations etc. As the authors rightly state the reason why students lack the teaching and eventual learning of clinical examination skills are many, but most are related to time and lack of teachers and the conflict between service and teaching requirements, so adding technology enhanced learning I don't feel can improve this situation. The ability to use such technology is important however and making sure that our future junior doctors know how to use this technology is important so I would not write it off from any curriculum. I wonder if more and appropriate use could be made through their use in clinical anatomy.

Reviewer Expertise:

NA

No decision status is available

# Reviewer report: Reviewer response for version 1

**DOI:** 10.21956/mep.18983.r27204

**Published:** 2018-08-07

**Authors:** P Ravi Shankar

**Affiliations:** 1 American International Medical University

This review has been migrated. The reviewer awarded 3 stars out of 5 This is an interesting study. Physical examination skills were very important when I was in medical school. As pointed out by the authors the teaching of physical examination skills has declined. One reason could be the more widespread adoption of technology. Also due to various reasons some of which have been mentioned in the article the amount of time doctors spend with their patients has reduced. Combining ultrasound with physical examination has the benefit of providing real time feedback to students about their physical exam findings. The drawback may be that the dominance of technology as a diagnostic method is being highlighted. The evaluation portion of the manuscript still needs more work. There have been other studies about the use of handheld ultrasound devices in teaching various skills to students some of which could be discussed by the authors. This is a well-written and lucidly presented manuscript. I enjoyed reading the manuscript which would be of interest to clinician educators. I would also be interested in knowing the cost of the handheld ultrasound devices and I believe the cost of these devices has been declining.

Reviewer Expertise:

NA

No decision status is available

# Reviewer report: Reviewer response for version 1

**DOI:** 10.21956/mep.18983.r27202

**Published:** 2018-08-07

**Authors:** John Sandars

**Affiliations:** 1 Edge Hill University

This review has been migrated. The reviewer awarded 1 stars out of 5 The topic of developing clinical examination skills is important and the use of handheld ultrasound devices appears to be an innovative approach to supplement more 'traditional' methods of teaching and learning clinical examination skills.However, this study has several important limitations:- The previous use of handheld devices as an approach is not discussed and linked to previous studies. It is unknown as to the extent to which this study adds to what is already known and the extent to which the study is innovative.- The teaching and learning intervention is complex, with several components, i including the use of handheld devices. There is a clear description of the intervention and the components. However, the evaluation requires greater detail and clarification as to the contribution of each component and in combination. A clear rationale that considers perceptions of usefulness (with specific details) and usability (ease of use) is essential for wider transfer and uptake to other contexts.- An essential aspect of any educational intervention that is being considered for more widespread use are further details on important issues such as cost of devices, training of users and trainers, difficulties encountered during the intervention (e.g. sudden loss of battery power or patient refusal)

Reviewer Expertise:

NA

No decision status is available
